# Characterization of EN-1078D, a poorly differentiated human endometrial carcinoma cell line: a novel tool to study endometrial invasion in vitro

**DOI:** 10.1186/1477-7827-5-38

**Published:** 2007-09-25

**Authors:** Marie-Claude Dery, Celine Van Themsche, Diane Provencher, Anne-Marie Mes-Masson, Eric Asselin

**Affiliations:** 1 Département de Chimie-Biologie, Groupe de Recherche en Biopathologies Cellulaires et Moléculaires, Université du Québec à Trois-Rivières, C.P. 500, Trois-Rivières, Québec, G9A 5H7, Canada; 2 Département d'Obstétrique-Gynécologie/Département de Médecine, Université de Montréal; Institut du Cancer de Montréal; Centre Hospitalier de l'UdeM (CHUM) – Hopital Notre-Dame, Montréal, Québec, H2L 4M1, Canada

## Abstract

**Background:**

To date, tools to study metastasis in endometrial cancers are insufficiently developed. The aim of this study was to characterize the cell line EN-1078D, a new endometrial carcinoma cell line derived from a metastasis to the ovary.

**Methods and Results:**

Cells were characterized using cytology, transmission electron microscopy, karyotyping and morphological appearance in culture. Molecular features were determined by RT-PCR, Western Blot, FISH and sequencing. MTT proliferation assays were performed to investigate the sensitivity of EN-1078D to anticancer agents such as cisplatin and doxorubicin. Also, subcutaneous and intravenous injections in nude mice were done to test the tumorigenic and metastatic properties of EN-1078D cells. Our results indicate that EN-1078D cells express both oestrogen receptors isoforms (ER alpha and ER beta) and also low levels of progesterone receptor B (PR-B). In addition, this cell line expresses high levels of MMP-2 and MMP-14 mRNA, low levels of TIMP-1 and TIMP-2 transcripts and no detectable levels of MMP-9 mRNA. Moreover, all nude mice developed tumors by subcutaneous injections and cell invasion was observed in vitro in response to TGF-beta 3. Her-2/neu was not overamplified but mutations in the C-2 domain of PTEN gene as well as codon 12 of the K-Ras gene were found. Finally, EN-1078D shows sensitivity to drugs commonly used in chemotherapy such as cisplatin and doxorubicin: IC50 of 2.8 μM of cisplatin after 72 hours of exposure and 0.54 μM of doxorubicin after 48 hours.

**Conclusion:**

Taken together, these results suggest that EN-1078D will be an excellent tool to study the properties of metastatic endometrial cancer cells in vitro and their regulation by sex steroids.

## Background

Uterine cancer is the fourth commonly diagnosed cancer among women in the North America. Ninety-seven percent of all cancers of the uterus arise from the glands of the endometrium and are known as endometrial carcinomas [1]. When diagnosed at early stages of the disease, this type of cancer is a highly curable malignancy with a 5-year relative survival rate of more than 80% [2]. However, patients presenting metastases have a 5-year survival rate of less than 20% [2]. Indeed, metastasis represents the main cause of death for patients with endometrial carcinoma. Very few models are available, to date, for the experimental characterization of factors involved in the metastatic phenotype in endometrial carcinoma cells.

Cell models of endometrial cancers are characterized by particular gene expressions and mutations which stratefy the disease. The cytokeratins (Ker) and other types of intermediate filaments are routinely used as indicators of tumor cell types as well as markers of differentiation [3], because their composition in any particular epithelium is predictable [4] The presence of functional steroid receptors, estrogen receptor alpha and beta (ERα and ERβ), progesterone receptor A and B (PR-A and PR-B) has been quantitatively associated with histologic differentiation [5], response to therapy [6] and metastatic potential [7]. Sex steroid hormones influence the metastatic phenotype in cancer cells, notably by regulating adhesion/de-adhesion events, angiogenesis, cellular invasion into the basement membrane and interstitium [8]. Therefore, the expression levels of ER and PR, as well as the impact of sex steroids are important regulators of endometrioid cells.

The uterus undergoes extensive tissue remodelling throughout each reproductive cycle and these dynamics change are regulated, in part, by the matrix metalloproteinase (MMP) system [9]. The MMPs are a family of proteolytic enzymes that can cleave a large array of extracellular matrix (ECM) proteins as well as other cellular, non-matrix proteins. Particular MMPs (including MMP-2, MMP-9 and MMP-14) are involved in key events in cancer cells, including proliferation, apoptosis and angiogenesis [10,11]. Importantly, loss of control of MMP activity has been linked to the malignant potential of tumors by enhancing invasion and metastasis [12]. When present in sufficient amount, the tissue inhibitors of MMPs (TIMPs) specifically inhibit MMP activity [11]. These interplay of these molecules are critically important in tumor metastasis.

Mutations in particular oncogenes and tumor suppressor genes can promote cancer cell development and their characteristic profile can help identify cancer cell types. Mutations in the tumor suppressor phosphatase and tensin homologue deleted on chromosome Ten (PTEN) gene can been found in approximately 50% of endometrial cancer cells [13-15]. Mutation in both PTEN gene alleles results in the expression of an inactive PTEN protein, which can not prevent activation of activated-by-kinas-tyrosine protein also called Akt and results in constitutively active Akt pathway. Since active Akt blocks the action of several pro-apoptotic proteins [16], apoptosis is deregulated in mutated PTEN cells. Mutational activation of K-ras (Kirsten rat sarcoma) has been observed in 10–30% of endometrioid carcinoma [17]. Ras is a signal transducer located on the inner surface of the plasma membrane. Hyperexpression of *ras *results in growth stimulation, whereas point mutations at codons 12, 13, and 61 alter its structure, preventing inactivation and causing cell transformation [18]. Amplification of Her-2/neu, a proto-oncogene with a high degree of homology to the epidermal growth factor (EGF), is associated with local invasion and tumor progression of endometrial carcinoma [19-22]. As these molecular alterations are hallmarks of endometrioid cancers, their characterization in cellular models is important. We had the opportunity to characterize a new endometrial cell line that was recently derived from an ovarian metastase, which represents a putative new tool for the study of endometrial carcinoma metastasis *in vitro*. We have extensively characterized this cell line in term of growth and molecular properties.

Finally, deregulated apoptotic mechanisms that allow cancer cell to proliferate and metastasize can lead to cancer cell resistance to pro-apoptotic agents such as chemotherapeutic drugs. We have characterized the response of EN-1078D cells to the main chemotherapeutic agents currently used for the treatment of endometrial cancer, cisplatin and doxorubicin. Together, this information can be applied to cellular and molecular studies in EN-1078D.

## Methods

### Patient data

The donor of the EN-1078D cell line was a 52-year-old woman, gravida 1, para 1, menopaused since two years. Histopathological examination revealed an endometrial adenocarcinoma poorly differentiated stage IIIC, with ovarian and ganglionic metastasis. At the time of surgery, the advanced tumoral invasion of the uterus did not allow its removal and bilateral ovariectomy was done. The patient was initially treated with megestrol acetate (160 mg/day) associated with local radiotherapy (45 Gy) and endocervical curietherapy. Six years after the initial surgery, the patient is still alive without recurrence.

### Histology of the original tissue and cell line establishment

The cell line was obtained from a patient of Centre Hospitalier de l'Université de Montréal and isolated from a metastase which completely replaced the ovaries. Informed consent was obtained and research studies approved by the Montreal University Institutional Review Board. The ovarian tumor consisted mainly of cells in Indian file with some glandular structures and central necrosis typical of an endometrial biopsy. At histopathological examination, immunohistochemistry analysis revealed 80% positivity staining for oestrogenic receptors and 25% for progesteronic receptors. Cells were established in culture as described previously [23]. Briefly, tumor tissue was minced with scissors into 2–4 mm explants in OSE media (without Fetal Bovine Serum (FBS)). Enzymatic dissociation was accomplished by digestion with collagenase and aggregates were dissociated by gently pipetting. The cellular fraction was diluted 1:5 in OSE media supplemented with 10% v/v FBS and incubated undisturbed at 37°C in 5% CO_2_/air for 24–48 hours. After cells had adhered to the plastic, they were washed once in PBS and subsequently passaged in OSE/10% v/v FBS until the cell line was stably established. Afterwards, the cells were grown and maintained in DMEM-F12 with HEPES (4-(2-hydroxyethyl)-1-piperazineethanesulfonic acid), and the medium was supplemented with 10% v/v bovine growth serum (BGS) and 50 μg/ml gentamycin. Until now, the cell line was subcultured more than a 100 passages and is preserved under cryogenic conditions (5% v/v DMSO (dimethyl sulfoxide)/culture media, in liquid nitrogen). Experiments using this cell line were carried out after long term passage (>76 passages). For experimentation without steroid hormones, the cell line was cultured at least two weeks in phenol-free DMEM-F12 supplemented with 10% v/v dextran-charcoal stripped FBS (FBS DC) and 50 μg/ml gentamycin, prior experimentation.

### Morphology of the cultured cells

Cells grown in culture flasks were photographed by phase-contrast microscopy. For transmission electron microscopy study, the cells were grown to confluence in culture flasks. They were washed with 0.1 M cacodylate buffer (pH 7.4) and fixed with 2.5% v/v glutaraldehyde in cacodylate buffer for 1 hour at room temperature. After washing twice with buffer, the cell layer was delicately detached with the help of cell scraper (Costar, VWR Canlab, Mississauga, ON, CA) and was postfixed with 1% v/v osmic acid for 1 hour at room temperature. The cells were then washed in cacodylate buffer, dehydrated in graded concentrations of ethanol and embedded with Spurr overnight. After polymerization, thin sections were contrasted with 4% w/v uranyl acetate in 50% v/v methanol following by lead citrate coloration. Specimens were examined and photographed with a Phillips EM 208S electron microscope.

### *In Vitro *growth assays

Growth curves were performed by seeding cells (1 × 10^5^) into 42 flasks (25-cm^2^) in fresh culture medium (reference day 1). From day 2 to day 15, total cell numbers of 3 flasks were counted with a hemocytometer and discarded thereafter [24]. The media was changed every 3 days. The population doubling time was calculated from the slope of the growth curve during the logarithmic phase while saturation density was determined by the plateau phase. For plating efficiency studies, one hundred cells were plated in 100-mm petri dishes. After two weeks, the colonies formed were fixed in 95% v/v ethanol and stained with methylene blue. Plating efficiency was determined by the ratio of the number of colonies (more than 50 cells) to the total number of inoculated cells [25]. The experiments were done at each ten passages and were repeated ten times.

### *In vivo *growth assays

Subcutaneous tumor xenografts were established in four 6-week-old nude mice (Charles River Laboratories, Lasalle, Qc, CA) by injection of 1 × 10^6 ^EN-1078D cells in 100 μL of 2 mg/mL Matrigel. (VWR, Mississauga, ON, CA). The mice were injected at both flanks near the posterior legs. The day following inoculation, mice received a subcutaneous injection of 17 β-estradiol (E_2_) (0.15 mg/animal in 5% cremophor and 5% ethanol saline solution) to stimulate cell proliferation. Tumor size was measured once a week using calipers. Tumor volume was calculated using the formula 0.5 × length × (width)^2 ^[26]. Eight weeks after injection, mice were killed and tumors were harvested. The tumors were fixed in 10% v/v formalin solution and embedded in paraffin. Tumor sections (7 μm) were mounted on polylysine-coated slides, deparaffinized, rehydrated, and then stained with haematoxylin.

### Invasion assay

Cells were treated or not with Transforming growth factor-beta3 (TGF-β3) (10 ng/ml) for 24 hours and then trypsinised and washed in fresh media with serum. Cells were then resuspended in media without serum and counted. 3 × 10^4 ^cells were plated in a Transwell^® ^Permeable support (Costar 3432, Corning, USA) with a 8.0 μM pore size polycarbonate membrane. Prior to cell plating, a layer of BD Matrigel Low™, diluted 1:5 in fresh media without serum was prepared and solidified. Fresh media with 10% BGS was put in the well below the support. Cells were cultured for 24 hours. At the end of the assay, the cells which had adhered in the bottom of the well and/or in filter were counted. These experiments were repeated 3 times.

### Cytogenetic analysis

Repeated chromosome analyses were carried out at each ten passages to examine the *in vitro *chromosomal evolution of this cell line. A total of 160 metaphases spreads were photographed and the chromosome numbers in each spread were counted. Harvesting, fixation, R and G-banding of the chromosomes were induced by using classical techniques in cytogenetic. To confirm chromosome X and copy number of the Her-2/neu gene, we performed fluorescence *in situ *hybridization (FISH) with painting probes CEP X (DXZ1)/Y (DYZ1) alpha satellite III and Her-2 IVD Kit (Vysis/Abbott Laboratories, Mississauga, ON, CA) according to the protocols supplied by the company and analyzed the karyotype with Cytovision.

### MTT proliferation assays

For drugs assay, cells were plated at a density of 1.5 × 10^4 ^cells/well in 96-wells plates, 24 hours before the assay. Cells were cultured for 24, 48 and 72 hours in the presence of increasing concentrations of cisplatin and doxorubicin (0; 0.625; 1.25; 2.5; 5; 10 and 20 μM in DMF (dimethylformamide)). HeLa, a cervical cancer cell line, which we have previously found to be sensitive to these drugs was used as positive control, while KLE, an endometrial adenocarcinoma cell line poorly inhibited by the drug, was used as negative control. For steroid hormones assays, cells cultured in phenol-free media were plated at a density of 2 × 10^4^cells/well in 96 wells plate, 24 hours before the assay. Preliminary dose-response assays were done to determine the treatments conditions: cells were cultured for 48 hours with 10^-6^M of progesterone (P_4_) and for 24 hours with 10^-7^M of E_2_, supplemented with 1% FBD DC. At the end of the culture period, 10 μl of MTT (5 mg/ml, thiazolyl blue tetrazolium bromide) was added to each well. After 4 hours of incubation with MTT, 100 μl of solubilization solution was added (10% w/v SDS in 0.01 M HCl) and the microplate was incubated overnight (37°C, 5% CO_2_/air). The OD (620 nm) was read with the Fluostar Optima reader. The experiments were repeated 3 times.

### Semi-quantitative RT-PCR analyses

In order to measure the presence or absence of transcripts for keratins, oestrogen and progesterone receptors, vimentin and desmin, semi-quantitative RT-PCR analyses were performed. Primers (Invitrogen, Burlington, ON, CA) chosen are described in Table 1. Conditions were optimized by testing different primer concentrations and different number of cycles to avoid near-plateau or saturated reactions. Total RNA (0.2 μg/μl) was used for preparation of first strand cDNA by reverse transcriptase (RT). The RNA samples were incubated (65°C, 10 min) with 2:1 oligo dT (deoxythymidine) primers in a final volume of 10:1. Samples were then incubated (37°C, 60 min) in 20:1 of a reaction buffer 10× containing dithiothreitol (DTT, 100 mM), deoxynucleotide triphosphate (dNTPs, 5 mM) and Muloney Murine Leukemia Virus Reverse Transcriptase (MMLV-RT, 200 U). The reaction volumes were brought up to 60:1 with autoclaved water. A negative control was also included to control for contaminating genomic DNA in the RNA template.

**Table 1:** PCR primers, cycling conditions and positive controls used.

**Primer**	**Sens Primer**	**Antisense Primer**	Size (bp)*	**annealing (celsius)**	**cycles**	**extension**	**positive control**
Keratin 1	5'-gatgaaggccacggtgatca-3'	5'-gacttgagttggggtgccta-3'	627	64	36	1 min	KLE
Keratin 4	5'-ctccagcaaaaaccttgagc-3'	5'-aagtcattctcggctgctgt-3'	186	58	36	30 sec	KLE
Keratin 5	5'-tctcgccagtcaagtgtgtc-3'	5'-atagccacccactccacaag-3'	247	58	36	30 sec	KLE
Keratin 7	5'-caggatgtggtggaggactt-3'	5'-ttgctcatgtaggcagcatc-3'	116	58	30	30 sec	KLE
Keratin 8	5'-agatgaaccggaacatcagc-3'	5'-tccagcagcttcctgtaggt-3'	262	58	30	30 sec	KLE
Keratin 13	5'-gtcttcagcacccagaggag-3'	5'-ttgcagaaaggcaggaaact-3'	246	58	36	30 sec	Hec-1-A
Keratin 18	5'-cacagtctgctgaggttgga-3'	5'-gagctgctccatctgtaggg-3'	164	58	36	30 sec	KLE
Keratin 19	5'-tttgagacggaacaggctct-3'	5'-gccatgacctcatattggct-3'	275	58	30	30 sec	KLE
Keratin 20	5'-acgccagaacaacgaatacc-3'	5'-acgaccttgccatccactac-3'	198	61	36	30 sec	KLE
Vimentin	5'-gagaactttgccgttgaagc-3'	5'-tccagcagcttcctgtaggt-3'	170	58	36	30 sec	KLE
Desmin	5'-caagctgcaggaggagattc-3'	5'-ggcagtgaggtctggcttag-3'	241	62	36	30 sec	KLE
ER alpha	5'-gtgcctggctagagatcctg-3'	5'-agagacttcagggtgctgga-3'	265	66	36	30 sec	MCF-7
ER beta	5'-tcaggcatgcgagtaacaag-3'	5'-gcttttactgtcctctgccg-3'	167	65	36	30 sec	MCF-7
PR-A	5'-gcttcaagttagccaagaagagt-3'	5'-ctggaaattcaacactcagtg-3'	290	58	36	30 sec	Ishikawa
PR-B	5'-acaccttgcctgaagtttcg-3'	5'-tccaagacactgtccagcag-3'	160	64	35	30 sec	Ishikawa
MMP-2	5'-aggcaagtggtccgtgtgaa-3'	5'-acagtggacatggcggtctcag-3'	369	66	36	30 sec	KLE
MMP-9	5'-caacatcacctattggatcc-3'	5'-cgggtgtagagtctctcgct-3'	479	60	36	30 sec	KLE
MMP-14	5'-ccagggtctcaaatggcaaca-3'	5'-ccatggaagccctcggcaaa-3'	219	66	36	30 sec	**
TIMP-1	5'-accagaccaccttataccagcg-3'	5'-ggactggaagcccttttcagag-3'	395	58	36	30 sec	RL-95-2
TIMP-2	5'-atgcagatgtagtgatcagggc-3'	5'-gatgaagtcacagagggtgatg-3'	272	58	36	30 sec	RL-95-2
β-actin	5'-gaggatcttcatgaggtagtctgtcaggtc-3'	5'-caactgggacgacatggagaagatctggca-3'	348	58	25	30 sec	none

* Size of amplified fragment; ** PMA-treated THP-1 cells

Each reaction mixture (final volume 50:1) contained 1× Buffer, RT template or negative control (5:1), MgCl_2 _(50 mM), dNTPs (5 mM), primers (pM; 2,5:1 each) and Taq polymerase (5 U/μl). The PCR cycling conditions chosen were 30 sec at 94°C, 30 sec at the appropriate annealing temperature and extension time at 72°C (Table 1), followed by a 5 min extension at 72°C. Reaction products were analysed on 1% w/v agarose gels. Bands were visualized by ethidium bromide staining. β-actin was used as loading control for each experiments.

### Protein extraction and Western analysis

Cells were trypsinized, lysed in lysis buffer (PBS 1× pH 7.4; 1% Nonidet P-40; 0.5% Sodium deoxycholate; 0.1% SDS; Protease Inhibitor Cocktail Tablets (Roche, Indianapolis, IN, USA), frozen and thawed three times, and centrifuged (15,700 g, 20 min at 4°C) to remove insoluble material. Supernatant was recovered and stored at -20°C pending analysis. Protein content was determined with the Bio-Rad DC Protein Assay (Bio Rad, Mississauga, ON, CA). Protein extracts (50 μg except for cytokeratines where 25 μg was used) were heated (95°C, 3 min), resolved by 8, 10 or 14% w/v SDS-Polyacrylamide gel electrophoresis (SDS-PAGE) and electro-transferred to nitrocellulose membranes (15 V, 30 min) using a semi-dry transfer apparatus (Bio-Rad). Membranes were then blocked (1 hr, RT) with PBS 1× containing 5% w/v non-fat milk powder, and then incubated with primary antibody (ERα: LabVision Ab15, dilution1:500, positive control MS-1071-PCL; ERβ: Labvision beta Ab-24 1:2000, positive control RB-037-PCL; PRA-B: Cell Signaling #3172 dilution 1:1000; Pan-cytokeratins: Sigma c-2562, dilution 1:75000) overnight (except for β-actin, one hour) at 4°C, and subsequently with Horseradish peroxidase (HRP)-conjugated anti-rabbit secondary antibody (1:4000; RT, 45 min) or with HRP-conjugated anti-mouse secondary antibody (1:2500; RT, 45 min). Peroxydase activity was visualized with the Super Femto kit (Pierce/Fisher, Nepean, ON, CA), according to the manufacturer's instructions. β-actin was used as a loading control for each experiments (Sigma A-3854 clone AC-15, dilution 1:60000, one hour).

### Sequencing of the PTEN and K-ras genes

Mutation of the PTEN and K-ras gene at codons 12/13 and 61 respectively were examined by sequencing early passage EN-1078D. PTEN and K-ras gene were amplified by PCR using the following primer pairs: for PTEN, 5'-CCCAGACATGACAGCCATC-3' (forward) and 5'-TTTCATGGTGTTTTATCCCTCTT-3' (reverse); for K-ras, 5'-AGGCCTGCTGAAAATGACTG-3' (forward) and 5'-TCCTGAGCCTGTTTTGTGTCT-3' (reverse). An initial denaturation of 3 min at 94°C was followed by 35 cycles of 30 sec at 94°C, 30 sec at 58°C for K-ras/64°C for PTEN and 30 sec at 72°C for K-ras/1 min 30 for PTEN, and a final elongation step of 5 min at 72°C. PCR products were cloned in competent *E. coli *using pcDNA3.1/V5-His TOPO TA expression kit (Invitrogen, Burlington, On, Ca) according to the manufacturer's instructions. For sequencing, PCR products were purified with Miniprep purification kit (Qiagen, Mississauga, ON, CA). The sequencing was carried out using automatic sequencers models capillary ABI PRISM 3100 and ABI PRISM 377 in the Laboratory of Synthesis and Analysis of Nucleic Acids at Laval University (Québec, QC).

## Results

### Cell morphology and growth

**Figure 1:**
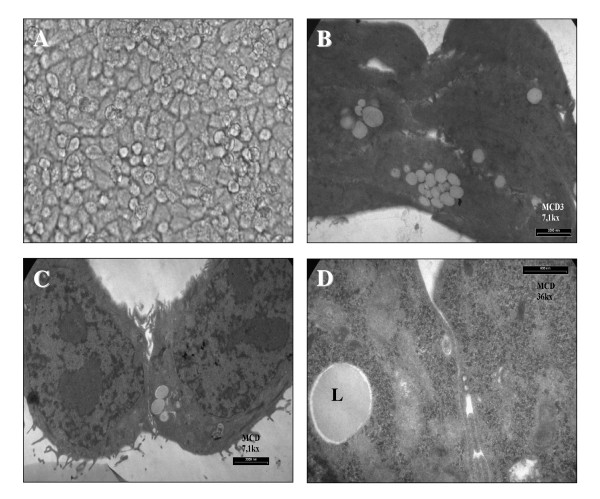
A) Phase contrast features of the monolayer-cultured EN-1078D cells revealing a sheet of polygonal cells with a pavement-like arrangement. B) Transmission electron microscopy showing the tendency of cells to pile up. C) Ultrastructural aspects of EN-1078D cell line by transmission electron microscopy. Cultured cells show a high nucleus-cytoplasmic ratio, euchromatic nuclei, prominent nucleoli and well-developed microvilli. D) Higher magnification shows intracytoplasmic lipid droplets (L), and exhibits intricate cytoplasmic interdigitations but no desmosomes were observed.

Early-passage EN-1078D was examined at low and high resolution. Light microscopy revealed small, polygonal cells organized in a pavement-like arrangement (Fig. 1A). The cell line showed no contact inhibition and was characterized by a tendency to pile up (Fig. 1B). The transmission electron microscopy revealed the presence of numerous well-developed microvilli, intracytoplasmic lipid droplets but no desmosomes (Fig. 1C). Only intricate cytoplasmic interdigitations were observed (Fig. 1D).

**Figure 2:**
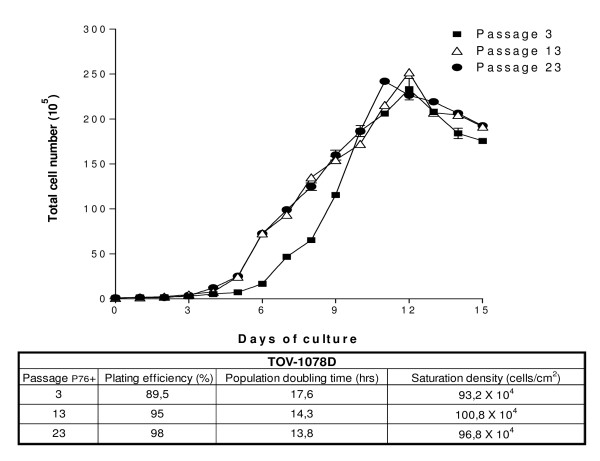
Growth curves and characteristics of EN-1078D.

To address the *in vitro *growth properties of EN-1078D, cells were seeded into culture dishes and counted every 24 hours (Fig. 2). Results showed logarithmic growth for the first 12 days, and we observed that cell growth continued even when cells reached confluency and seemed limited by the exhaustion of the culture media. Presence of floating cells was noted from the day 12 of the experiment but no layer of cells detached from the flasks even when cells reached confluency. The plating efficiency increased (89.5% to 98%) and the population doubling time decreased (17.6 to 13.8 hours) with increasing number of passages.

### Molecular characterization of EN-1078D

**Figure 3:**
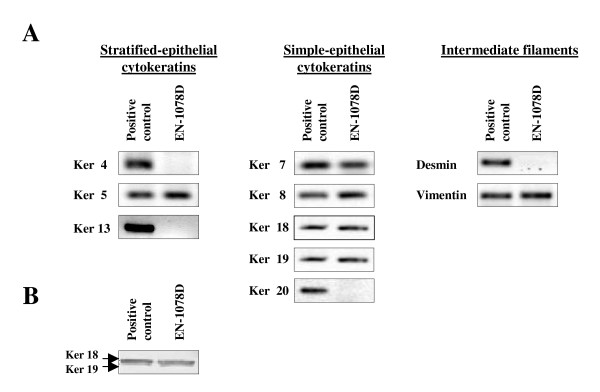
Expression of cytokeratins in EN-1078D. A) RT-PCR analysis for stratified-, simple-epithelial cytokeratins and for the other types of intermediate filaments. In EN-1078D cells, the simple-epithelial cytokeratins are predominantly expressed. Vimentin expression is consistent with the neoplasic tissue while absence of the expression of desmin confirmed that EN-1078D is not an endometrial sarcoma. B) Expression of cytokeratins 18 and 19 by Western Blot analysis KLE was used like as positive control Data shown are representative of results obtained from three independent assays.

To verify the epithelial origin of the cell line, we profiled the expression of cytokeratins. RT-PCR (Reverse-transcriptase polymerase chain reaction) revealed a predominance of the simple epithelial cytokeratins as indicated by the presence of mRNA for cytokeratins 7, 8, 18 and 19 (Fig. 3A). Western Blot analysis confirmed the expression of cytokeratins 18 and 19 (Fig. 3B); no suitable antibody was available to confirm the expression of Ker7 and Ker8 proteins. Stratified-epithelial cytokeratin 5 mRNA was also detected (Fig. 3A). We also examined the expression of other intermediate filaments: as expected, the transcript of a marker of muscle cells, desmin, was not detectable, but mRNA from vimentin, which is overexpressed in cancerous cells, was present (Fig. 3A).

**Figure 4:**
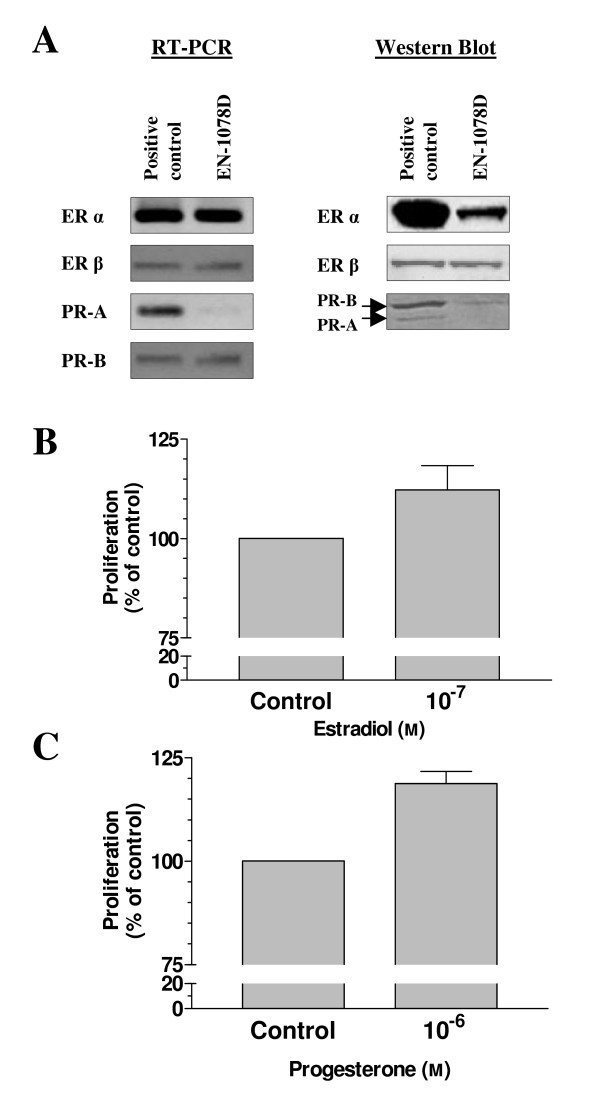
Sex steroid receptor status. A) Both estrogen and progesterone receptors mRNA are detected by RT-PCR but the signal for PR-A is very weak and protein is not detected by Western blot analysis. Ishikawa cell line was used as a positive control for PRA-B antibody. Data shown are representative of results obtained from three independent assays. MTT proliferation assay was conducted to verify the functionality of these receptors in EN-1078D cell line: B) 24 hours of exposure to 17β-oestradiol increases cell growth and C) a significant increase in proliferation was observed after 48 hours of exposure to 10^-6^M for progesterone (p < 0.05). Data shown are representative of results obtained from three independent assays.

EN-1078D cells were also tested for the presence of estrogen and progesterone receptors. Figure 4A shows the hormonal receptor status of the cell line. RT-PCR and Western Blot revealed the presence of both estrogen receptor alpha and beta. Only low levels of PR-A mRNA and protein are detectable although the EN-1078D cells expressed PR-B mRNA and protein. In an attempt to determine if these steroid hormones receptors were functional, we have subjected EN-1078D cells treated with increasing concentrations of 17β-estradiol and progesterone in phenol-free conditions to MTT proliferation assay. Both estradiol and progesterone could induce the proliferation of EN-1078D cells (Fig. 4B and 4C). This indicates that these receptors are functional in EN-1078D and also suggests that the properties of these cells can be modulated by sex steroids.

**Figure 5:**
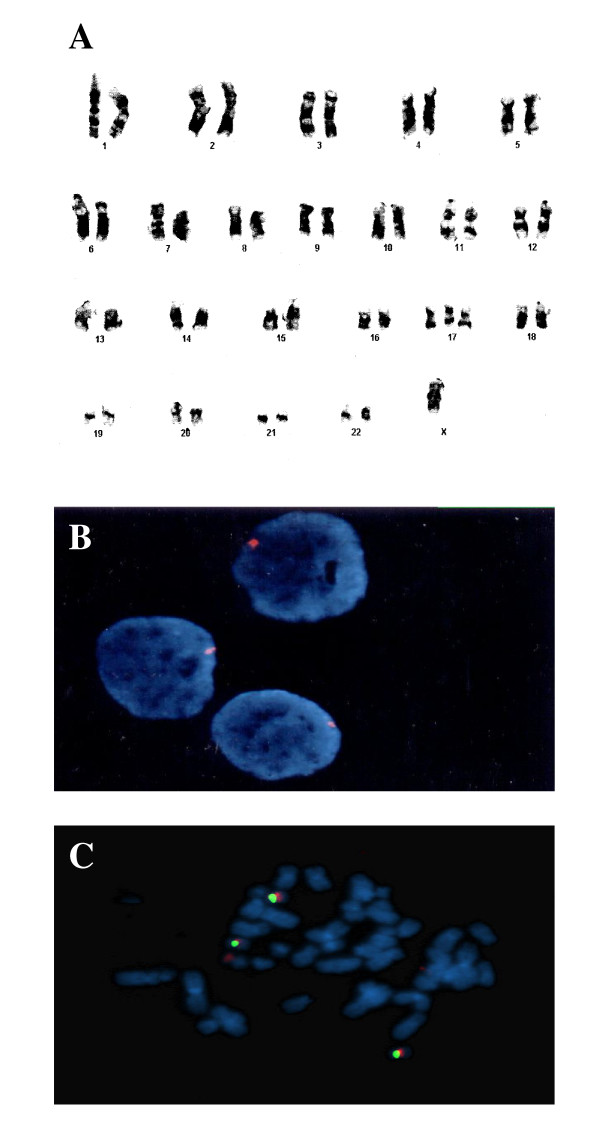
A) Karyotype analysis of EN-1078D (P76+13 passages), showing that all cells have monosomy of X chromosome. Most of the cells have trisomy of chromosome 17. B) FISH analysis of copy number of X chromosome with Probe CEP X (DXZ1) alpha satellite III. Probe DXZ1 marked in orange spectrum hybrid satellite DNA localized in the centromere of the X chromosome. Only one X chromosome per cell was observed. C) Metaphasic analysis of Her2/neu (human epidermal growth factor receptor 2) by fluorescence *in situ *hybridization (FISH). The HER-2 probe spans the entire HER-2 gene is labeled in spectrum orange. The CEP 17 probe is labeled in spectrum green and hybridizes to the alpha satellite DNA located at the centromere of chromosome 17 (17p11.1-q11.1). Inclusion of the CEP 17 probe allows for the relative copy number of the HER-2 gene to be determined.

In addition, we characterized the presence of chromosomal alterations in EN-1078D cells. Chromosome analysis revealed an aneuploid population with a complete monosomy for the sexual chromosome X in more than 90% of the cells examined (45, X). The presence of a minor population of cells was observed containing 46 chromosomes with a monosomy for the sexual chromosome and a trisomy for the chromosome 17 (46, X-X, +17) (Fig. 5A). The proportion of this second population varied with increasing number of cell passages, from 10% at first passage to 48% after 20 passages.

To confirm the chromosomal complement 45, X, two probes were used: CEP X (DXZ1) and Y (DYZ1) alpha satellite III. The DXZ1 probe, with an emission wavelenght in the orange spectrum, hybridizes to the satellite DNA localized in the centromere of the X chromosome, while the DYZ1 probe, with an emission wavelenght in the green spectrum, hybridizes to the satellite DNA III localized in Yq12 locus (Fig. 5B). The results showed only one positive signal in each 988 interphasic cells analyzed. No green signal was obtained indicating that Y chromosome was not present. Figure 5C shows an interphasic hybridization carried out with a probe located on the long arm of chromosome 17q11.2q12 called Her-2/neu. To determine the number of copies of the Her-2/neu gene present in this cancer cell line, we used a probe marked with a fluorescent protein emitting in the orange spectrum which covers the complete Her-2/neu gene as well as a probe marked with fluorescent protein emitting in the green spectrum hybridizing to the satellite DNA in the centromere of chromosome 17. The analysis of 1000 cells showed that only one copy of this gene was present in chromosome 17, indicating no amplification of Her-2/neu gene in EN-1078D cells: a low level of amplification range is between 2 and 4 signals/centromere [27]. We have found, however, other types of mutations in this cell line: sequencing revealed two sites of mutations in PTEN gene. Codon 275 is mutated by addition of a nucleotide and codon 367 replaces a valin for isoleucin. In addition, K-Ras gene analysis showed the classical G to T transversions in the codon 12 (data not shown).

### Chemosensitivity of EN-1078D cell line

**Figure 6:**
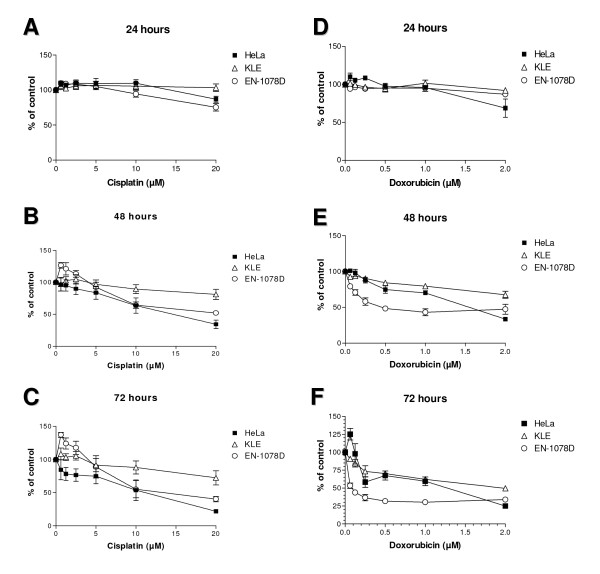
Determination of EN-1078D sensitivity to cisplatin (left panel) and doxorubicin (right panel). A, D) 24 hours of exposure to compounds B, E) 48 hours and C, F) 72 hours. Both Hela and EN-1078D are sensitive to cisplatin after 24 hours in contrast to KLE, which is chemoresistant. The three cell lines seem not to be affected by doxorubicin after exposure of 24 hours. KLE is more resistant than other cells lines for both 48 and 72 hours. Data shown are representative of results obtained from three independent assays.

We have examined the resistance of EN-1078D to two chemotherapeutic agents currently used for the treatment of endometrial carcinoma, cisplatin and doxorubicin, using the MTT proliferation assay. Two other cell lines were used as a reference: HeLa, a cervical cancer cell line that we showed to be sensitive to these anticancer agents, and KLE, an endometrial cancer cell line known to be chemoresistant [28]. Even after a short exposure of 24 hours to cisplatin, EN-1078D cells were more sensitive than both Hela and KLE (Fig. 6A–C). EN-1078D cells reached IC50 at 2.8 μM of cisplatin after 72 hours of exposure comparatively at 6.7 μM for HeLa while KLE cells showed 30% of growth-inhibition in the same conditions. In the case of doxorubicin, 48 h of exposure were required for EN-1078D cells to be growth-inhibited and reached IC50 to 0.5 μM of this compound; HeLa and KLE cells were more resistant to the effects of doxorubicin than EN-1078D cells (Fig. 6D–F) with a IC50 of 0.6 μM for HeLa after 48 hours of exposition and for 1.4 μM for KLE after 72 hours. Altogether these results indicate that EN-1078D cells are sensitive to the cytotoxicity of cisplatin and doxorubicin (Fig. 6F).

**Figure 7:**
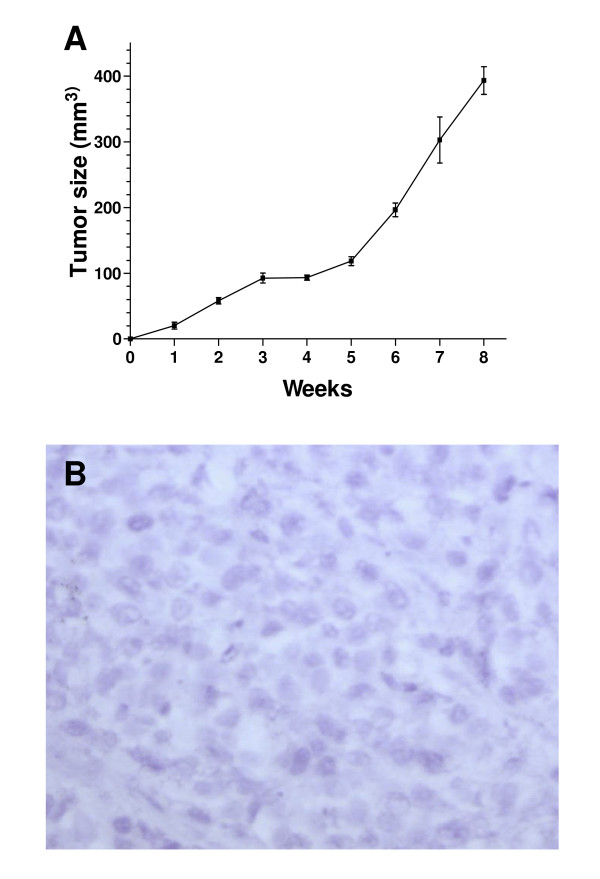
A) Tumorigenicity of EN-1078D cells in nude mice. B) Histology of subcutaneous tumor taken from nude mice showed large solid masses composed of poorly differentiated carcinoma cells with no apparent structures (haematoxylin, 40×)

**Figure 8:**
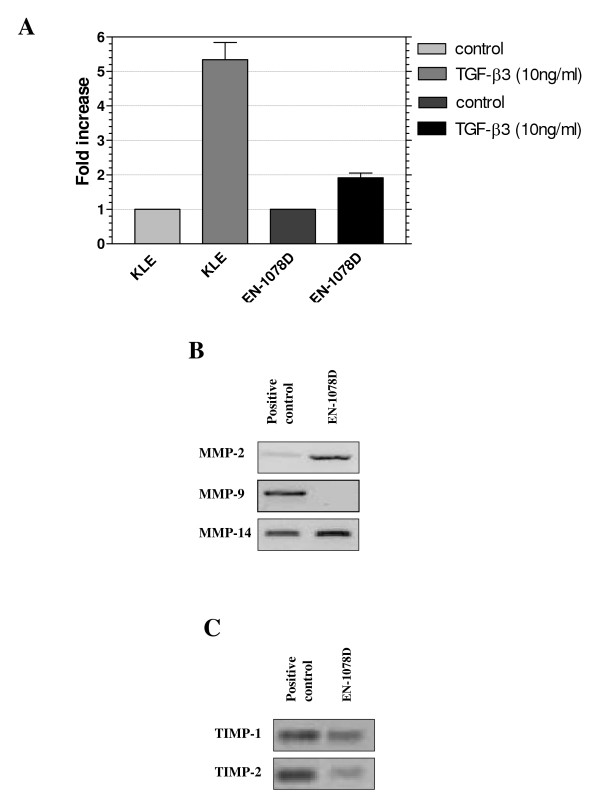
Invasion assay and expression of MMPs and TIMPs mRNA in EN-1078D. A) EN-1078D cells showed capacities to increase their invasiveness by 2 fold induction when they were stimulated with TGF-β3. B) The cell line expresses MMP-2 and 14 while MMP-9 was not detected. The signal for MMP-2 is stronger for EN-1078D cells than for the positive control used. C) Both TIMPs expressions were detected but TIMP-2 signal is weak. Data shown are representative of results obtained from three independent assays.

In order to test the tumorigenic potential of EN-1078D *in vivo*, we performed subcutaneous xenograft experiments in nude mice. All mice developed palpable tumors, and about 8 weeks after inoculation, the tumor size had reached a volume of 350 mm^3 ^or more (Fig. 7A). The encapsulated tumors were compact in periphery with a liquefied center. Histologic analysis of tumors showed no specific organisation pattern of tumor cells nor observable structures like basal lamina (Fig. 7B). These results indicate that EN-1078D cells are tumorigenic and can therefore be used for *in vivo *tumor growth experiments. No macroscopic metastases were visible in spleen, lungs or liver up to eight weeks post-xenograft. On the other hand, EN-1078D showed 2 fold increase of total number of the invasive cells when the cells were treated with TGF-β3[29] (fig. 8A) after 24 hours of invasion assays. Together, these results confirm that EN-1078D cells constitute an excellent *in vitro *model to characterize the molecular and cellular mechanisms involved in the invasive phenotype of endometrial carcinoma cells.

Constitutive expression and activity of MMPs increases the invasiveness of various types of cancer cells, and we have determined characterized the expression of MMP-2, MMP-9 and MMP-14 as well as TIMP-1 and TIMP-2 mRNA in EN-1078D cells, using RT-PCR (Fig. 8A and 8B). We found elevated levels of MMP-2 and its physiological activator, MMP-14 mRNAs (Fig. 8B), and low levels of their inhibitor TIMP-2 mRNA (Fig. 8C). As expected, we found that MMP-9 mRNA was not expressed constitutively (Fig. 8A), suggesting that external stimuli, such as contact with matrix protein in Matrigel, for instance, are required to induce the expression of the enzyme. Low levels of TIMP-1 transcript were found in EN-1078D cells (Fig. 8C).

## Discussion

The present study describes the characterization of a new poorly differentiated endometrial carcinoma cell line, EN-1078D, isolated from a metastasis to the ovary. Although many endometrial carcinoma cell line have demonstrated their capacities to form tumors and/or metastases in nude mice (Hec-1-A, Hec-1-B, RL-95-2 and Ishikawa) [30-32], only few endometrial cancer cell lines derived directly from metastatic cancer cells (AN_3_CA and KLE) [33,34].

We have confirmed the origin of simple epithelial cells [35] for this cell line and the endometrial carcinoma phenotype of the metastatic tumor cells because the EN-1078D cell line was isolated from a ovary. In addition, EN-1078D analysis revealed the presence of two cell populations in this cell line which is often the case in cultures derived from tissues. The presence of only one chromosome X in both populations of EN-1078D cell line confirmed the same origin for these two populations. The differentiation in two types of cell populations by the achievement of the third chromosome 17 was the fact of malignant transformation which seems to give an advantage for *in vitro *growth to this cellular population compared to the other one [36,37]. In addition, we have tested the response of EN-1078D cells to two chemotherapeutic agents commonly used: cisplatin and doxorubicin. In fact, with his high FIGO (International Federation of Gynecology and Obstetrics) grade (III), his poor differentiation and his aggressiveness, it is surprising that this cell line is so sensitive to these compounds. It is suggested that the karyotypes of the cultured cells may undergo considerable changes *in vitro *and some of these changes may represent the malignant transformation process of the tumor cells *in vivo *[35,36].

Sex steroid receptors status is crucial in the development of endometrial cancer. EN-1078D cells show all functional sex steroid receptors except for PR-A, which is not expressed. Expression of PR-B alone, a strong regulator of proliferation [8,38], is only seen in tumor cells [39] and was a feature of high-grade tumors. A study by Fujimoto and Ichigo [40] has reported that in all metastatic lesions of uterine endometrial cancers, the expression of PR-A mRNA was suppressed and PR-B mRNA was dominantly expressed which are in correlation with our observations. Additionally, the presence of the both ERα and ERβ in EN-1078D cells confirm that this cell line will become a very interesting tool of study because the majority of endometrial cancer cell lines available express only one of these two receptors.

Loss of progesterone responsiveness in endometrial cancer [41] combined with the expression of some MMPs might be related to the metastatic potential of EN-1078D cells. The level expression of MMP-2, MMP-14 and TIMP-2 in EN-1078D cell line is consistent with an endometrial cancer with high invasive potential [42]. The team of Graesslin [43] has found a relation between low TIMP-2 expression and myometrial invasion, lymphovascular space involvement, and lymph node metastasis. MMP-9 is normally induced under conditions that require tissue remodelling [44] and is predominantly expressed by inflammatory cells of the stroma [45]. EN-1078D cells do not express MMP-9 in basal and normal culture conditions but they may produce the enzyme in other, more physiological, conditions. Given that this cell line express constitutive levels of MMP-2 and MMP-14, and that EN-1078D cells were highly invasive *in vitro*, this cell line represent an important tool for the characterization and the study of the molecular and cellular mechanisms regulating endometrial carcinoma cell invasion.

In endometrial cancers, frequents lesions were observed including K-Ras and PTEN mutations and Her-2/neu amplification [46]. Our finding that K-Ras is mutated in EN-1078D cell line is supported by many studies suggesting that the majority of mutations present in the codon 12 of K-Ras and G to T transversions were predominating in the North American population [47]. Mutations of the Ras oncogene results in autonomous cell growth [48] by enhancing estrogen and antiestrogen (tamoxifen)-induced transcriptional activity of the ER activation function [49,50]. A significant correlation was found between ER-dependent PR expression and activating K-Ras mutations suggesting that enhanced activity of the ER activation function by stimulating phosphorylation mediated through mutational activation of the Ras-MAPK cascade may be one mechanism of hormone independence of endometrial cancer [51].

PTEN gene is frequently mutated in endometrial cancer. We have identified to 2 sites of mutations in EN-1078D cells affecting the C-terminal domain. This domain, in which ≥ 43% of PTEN mutations occur, contains many important subdomains that are common to other signal-transducing molecules [52]. The C2 domain (amino acids 186–351) is associated with phospholipids-binding regions [53] and has been identified in many proteins involved in signal transduction and membrane localization [54]. The C-terminal tail also contains a sequence rich in proline, glutamic acid, serine and threonine (PEST sequence) (amino acids 350–375 and 379–396), which are critical for PTEN stability [55]. PEST sequences target proteins for short intracellular half-lives and protein degradation. Paradoxically, deletion of these regions leads to decreased protein expression versus the expected increase. Nonetheless, these studies point out that the PEST regions are necessary for PTEN stability and in EN-1078D cell line the PEST region contains one mutation. PTEN antagonizes the PI3K/Akt pathway by dephosphorylating PIP_3_(Phosphatidylinositol (3,4,5)-trisphosphate), resulting in a decreased translocation of Akt to cellular membranes and subsequent down-regulation of Akt activation. It has been shown that expression of PTEN in cells leads to decreased levels of phospho-Akt (active form), and, therefore, to increased apoptosis [56,57]. For example, RL-95-2 and Ishikawa endometrial cancer cells lines have a mutation in the PTEN gene and express high levels of phospho-Akt [58-60]. However, the mutated PTEN-cell line EN-1078D express weak levels of phospho-Akt (data not shown) which suggests that the simple mutation of PTEN in this cell line is not sufficient to induce constitutive Akt phosphorylation.

In conclusion, our results suggest that EN-1078D cell line is an endometrial carcinoma originating from simple epithelial cells. This tumorigenic cell line, positive for both estrogen receptor isoforms and progesterone receptor B, presents mutations in PTEN and K-Ras genes, one tumor suppressor and one oncogene frequently mutated in endometrial cancers. Moreover, EN-1078D expresses high level of MMP-2, no MMP-9 and a weak expression of TIMP-2. These cells express weak levels of phosphorylated Akt and show sensitivity to drugs commonly used in chemotherapy. EN-1078D cell line showed tumorigenic capacities *in vivo *and is a very aggressive cell line with high invasiveness *in vitr*o. EN-1078D will be a useful tool to study the mechanisms involved in the invasion of endometrial cancer cells, and their regulation by sex steroids.
